# Dental pulp lymphatic vessel dynamics during tooth development and pulp stimulation in rodents

**DOI:** 10.1111/iej.14244

**Published:** 2025-04-25

**Authors:** Kento Tazawa, Di Chen, Akira Fujimura, Philip D. King, Hajime Sasaki

**Affiliations:** ^1^ Department of Cariology, Restorative Sciences, and Endodontics University of Michigan School of Dentistry Ann Arbor Michigan USA; ^2^ Department of Pulp Biology and Endodontics, Division of Oral Health Sciences Graduate School of Medical and Dental Sciences, Institute of Science Tokyo (Science Tokyo; formerly Tokyo Medical and Dental University (TMDU)) Tokyo Japan; ^3^ Department of Microbiology & Immunology University of Michigan Medical School Ann Arbor Michigan USA; ^4^ Division of Dental Education, Department of Oral Medicine, School of Dentistry Iwate Medical University Iwate Japan

**Keywords:** dental pulp development, dental pulp stimulation, lymphatic system, prospero‐related homeobox 1 protein (Prox1), three‐dimensional imaging

## Abstract

**Aim:**

The anatomy and functions of lymphatic vessels (LV) in mammals remain poorly understood compared to the blood vascular system. In particular, whether or not LV exist in the dental pulp is still controversial. This study aims to identify the existence of LV in the mouse dental pulp using a Prox1 (Prospero homeobox 1)‐eGFP transgenic mouse model combined with a tissue‐clearing technique.

**Methodology:**

Mandible or mandibular first molars of Prox1‐eGFP mice were extracted, cleared for whole‐mount observation and imaged using confocal microscopy. Dylight 594‐lectin was injected intracardially to differentiate Prox1‐eGFP+ LV from the blood vascular network. To further determine if pulpal LV act as an interstitial fluid drainage system, we examined ink absorption in surgically exposed dental pulp of mandibular first molars.

**Results:**

At the early stage of tooth development, abundant Prox1‐eGFP+ LV distinct from lectin‐labelled blood vessels were present in a dental pulp. However, after the initiation of root development, the expression of Prox1‐eGFP in dental pulp decreased. In mature dental pulp, Prox1‐eGFP+ LV was scattered with a discontinuous lumen. In response to a non‐infectious transient pulp stimulation (TPS), the Prox1‐eGFP+ LV increased in number and diameter with continuous lumen reaching the apical foramen. Ink particles applied to exposed dental pulp are distributed throughout the dental pulp via interstitial spaces and vessel‐like structures. Histological evaluation revealed that ink particles were mainly present in the cell‐free zone. However, due to TPS, ink particles were taken up into Prox1‐eGFP+ LV.

**Conclusion:**

Our findings suggest the presence of LV in the mature dental pulp that contributes to fluid drainage in this tissue together with the extravascular pathway.

## INTRODUCTION

The lymphatic system, along with the blood circulatory system, is an essential circulatory system that plays an important role in tissue fluid homeostasis and immune surveillance (Petrova & Koh, 2020). Open spaces between lymphatic endothelial cells (LEC) in initial lymphatic vessels (LVs) enable the uptake of extravasated fluid, which is eventually returned to the blood circulation. Additionally, antigens and antigen‐presenting cells in tissues gain access to the LV system through these spaces, reaching lymph nodes where they induce adaptive immune responses (Oliver et al., 2020). Given their central roles in tissue fluid homeostasis and immunity, LVs are found in most tissues. However, in some tissues, such as the brain, central nervous system, bones, bone marrow and teeth, LVs were considered absent until recently (Aspelund et al., 2015; Berggreen et al., 2020; Breslin et al., 2018; Gerli et al., 2010).

The use of genetically modified mice that express fluorescent proteins such as GFP in LVs, combined with tissue‐clearing techniques, has brought new insights into the LV system by allowing the identification of LV without the need for conventional sectioning (Choi et al., 2011; Zhong et al., 2017). Specific expression of GFP in LV is achieved using a Prox1 (Prospero homeobox 1) promoter. Prox1 is a master transcription factor gene for LV specification and development and is a reliable lymphatic marker not expressed in endothelial cells of blood vessels (BVs) (Wilting et al., 2002). Three‐dimensional (3D) visualization of intact skeletal tissues has been technically challenging due to their calcified nature (Greenbaum et al., 2017). Recently developed tissue‐clearing methods have made it possible to observe target molecules in 3D and to understand their spatial distribution comprehensively (Jing et al., 2018; Tazawa & Sasaki, 2023). This new approach has revealed the presence of LVs in the brain, previously thought to be absent (Louveau et al., 2015). This has led to a growing momentum to reevaluate the presence of LVs in other tissues.

Dental pulp is a vascular‐rich soft tissue surrounded by hard tissues such as enamel and dentin. This hard tissue acts as a physical barrier against microbial invasion and chemical and mechanical irritation, but at the same time, this barrier adversely affects the control of tissue pressure, creating an environment of low compliance to changes in tissue pressure (Van Hassel, 1971; Yu & Abbott, 2007). To relieve increased pressure in dental pulp, a functional fluid transport system is required. However, the details of such a mechanism have not yet been elucidated. Given the function of LVs in the drainage of interstitial fluid in other tissues, several studies have attempted to determine if LV are also present in dental pulp that might fulfil a similar role. Approaches such as electronic microscopy (Marchetti & Poggi, 2002), enzyme histochemistry (Matsumoto et al., 2002) and immunohistochemistry (Wiśniewska et al., 2021) have been used, but no consistent conclusions have been reached.

The present study aims to identify and characterize the structure of a putative dental LV network and evaluate its function in pulpal interstitial fluid drainage using novel observation techniques.

## MATERIALS AND METHODS

**FIGURE 1:**
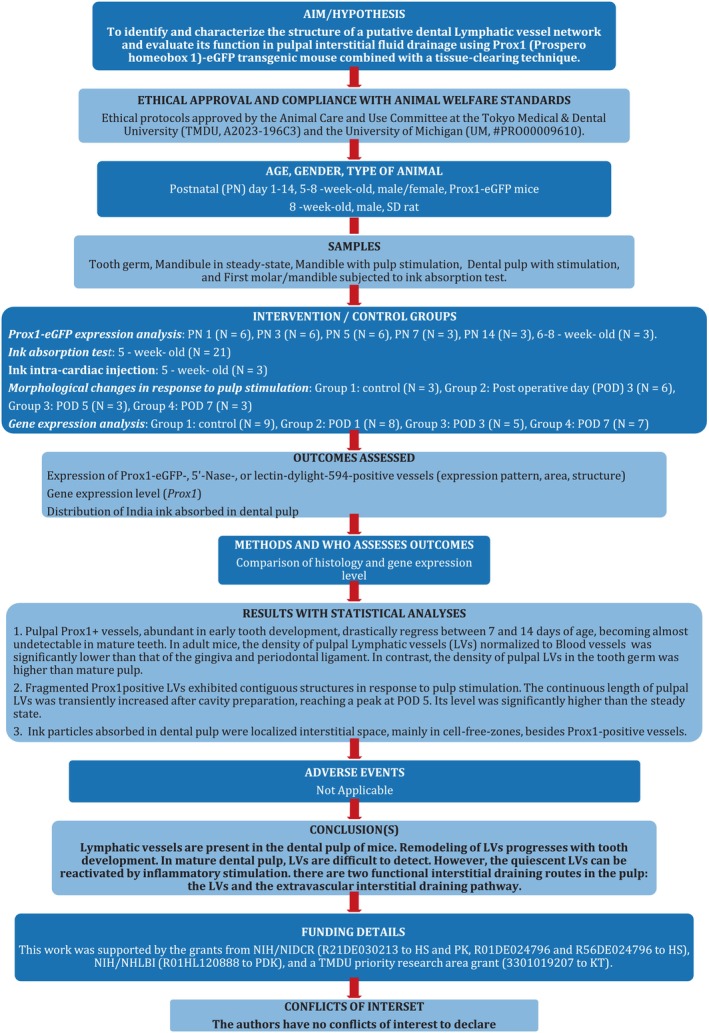
The flowchart illustrates the experimental design and analysis in animal studies conducted according to the PRIASE 2021 guidelines.

The manuscript of this animal study has been written according to Preferred Reporting Items for Animal Studies in Endodontology (PRIASE) 2021 guidelines (Nagendrababu et al., 2021). The steps involved in this study are shown in the PRILE flowchart (Figure 1).

### Animal experiments and tissue preparation

All animal studies were approved by the Animal Care and Use Committee at the Tokyo Medical & Dental University (TMDU, A2023‐196C3) and the University of Michigan (UM, #PRO00009610). Prox1‐eGFP transgenic mice (Tg(Prox1‐EGFP)221Gsat/Mmcd) (Choi et al., 2011) bred in‐house were subjected to two‐dimensional (2D) histological and 3D‐whole‐mount observations of the dental pulp. Animals were maintained by qualified animal caretakers under specific pathogen‐free conditions at the TMDU and the UM animal facilities. When animals were given invasive procedures, they were given sufficient sedation to prevent them from feeling pain. Once fully awake, the animals were returned to their cages and monitored regularly for abnormal behaviour.

### BV labelling with a vascular tracer

Under general anaesthesia (62.5 mg/kg ketamine HCl and 12.5 mg/kg xylazine in sterile PBS; intraperitoneal injection), mice received transcardial injection of Lycopersicon esculentum lectin conjugated to DyLight 594 (DL‐1177, Vector Laboratories, Newark, CA; weight ratio:100 μL/25 g, concentration: 1 mg/mL) for approximately 30 s. One minute after the injection, animals were subjected to perfusion fixation with phosphate‐buffered saline and 4% paraformaldehyde.

### A non‐infectious transient pulp stimulation (TPS)

Under general anaesthesia, TPS was given by shallow cavity preparation on the mesial surface of mandibular first molars using an electric handpiece (AEU‐178, Aseptico Inc., Woodinville, WA) with a 1/4 round bur (#14820, SS White Dental, Lakewood, NJ) under a surgical microscope (EVOLUTION XR6; Seiler, St. Louis. MO). The pulp was not exposed and not infected. This procedure is reported as an aseptically induced reversible pulpitis model; the pulpitis caused by a shallow cavity preparation resolves after 7 days, and the formation of reparative dentin is observed (Kieu et al., 2022). Animals with TPS were subjected to evaluation of Prox1‐eGFP signal and gene expression.

### 2D‐immunohistochemistry, 2D‐enzyme‐histochemical staining and 3D‐whole‐mount staining, and *Prox1* gene expression analysis

Mandibles and mandibular first molars isolated from Prox1‐eGFP mice were subjected to immunohistochemistry and 3D whole‐mount observation as described (Tazawa et al., 2020; Tazawa & Sasaki, 2023). Samples were fixed with 4% paraformaldehyde overnight and decalcified with 15% ethylenediaminetetraacetic acid for 1 week at room temperature. The thickness of the frozen section and whole‐mount sample is 14 μm and approximately 250 μm, respectively. To enhance the diminished eGFP signal after fixation, a rabbit polyclonal anti‐GFP antibody (1:500; NB600‐308; Novus Biologicals, Centennial, CO) was employed. The validity of this approach is demonstrated in Data S1. For tissue clearing, samples were subjected to the PEGASOS method. In brief, samples were decoloured in the decolourization solution for 2 days at 37°C. After three 1‐h washes in Tris‐buffered saline with Triton X‐100 (TBST), samples were subjected to immunolabelling. Samples were incubated in the blocking buffer for 24 h at room temperature and subsequently incubated with the anti‐GFP antibody prepared in the staining buffer for 4 days at room temperature. After three washes in TBST, the samples were incubated with a secondary antibody (Alexa Fluor 488‐conjugated anti‐rabbit antibody, ab150077; Abcam, Cambridge, MA) for 4 days at room temperature. Then, samples were thoroughly washed with TBST and placed in gradient tB delipidation solutions (30%, 50%, 70% v/v tB; each 24 h) for 72 h and then dehydrated in tB‐PEG for 2 days at 37°C. After dehydration, samples were immersed in the BB‐PEG medium at 37°C until the tissue became transparent.

For 5′‐nucleotidase (5′‐Nase) staining, mice were perfused with 0.1 M Cacodylate buffer (#AAA1813914, Thermo Fisher Scientific, Waltham, MA) containing 7% sucrose (#S0389, Sigma‐Aldrich, Burlington, MA) followed by 2% paraformaldehyde containing 1% calcium chloride. Mandibles were isolated and immersed in 4% paraformaldehyde containing 1% calcium chloride for 30 min, then washed with 0.1 M Tris‐maleate buffer (PH7.2, #T3128, Sigma‐Aldrich). After making frozen sections, sections were post‐fixed with 4% paraformaldehyde containing 1% calcium chloride for 30 min and applied with 5‐Nase reaction solution to the section at 37°C for 30 min. The colour was developed with 1% ammonium sulfide (# AA33286AE, Thermo Fisher Scientific).

To examine the effect of TPS on the dynamics of *Prox1* gene expression, male Sprague Dawley rats (a total of 29 rats, CLEA Japan, Tokyo, Japan) were subjected to TPS and euthanized on days 0 (control), 1, 3, and 7. Total RNA samples isolated from coronal dental pulps were reverse transcribed and subjected to reverse transcription polymerase chain reaction. The *Actb (b‐actin)* gene served as a reference gene.

### Ink absorption test

To examine the interstitial drainage pathway in mouse dental pulp, pulp exposure was prepared on the mesial cusp of the first molar without any bleeding under deep general anaesthesia. Then, India ink (Daiso, Tokyo, Japan) was applied dropwise (total 1 μL) to a dental pulp using pipette tips without any pressure. The total amount of ink absorbed by the pulp could not be accurately calculated because some ink spilit from the molar. After 10 minutes of ink absorption, mice received a transcardial injection of DL‐1177 as above and were euthanized by carbon dioxide inhalation. The mandibles and mandibular first molars were isolated, fixed, and subjected to 2D‐ and 3D‐observations. India ink was identified as black particles in specimens. The particle size of the pre‐measured India ink ranged from 80 to 800 nm (average 230 nm) using a Malvern Mastersizer 2000 (Malvern Panalytical, Malvern, UK). This ink absorption test was performed not only on healthy teeth but also on teeth subjected to TPS to examine the effect of the TPS on the function of pulpal lymphatics. Briefly, TPS was induced in the mandibular first molar as described in the TPS section; 3 days later, the pulp of the same tooth was exposed and the ink was applied as above.

### The quantitative analysis of tissue LV density and morphological changes

Tissue LV density was assessed as the ratio of eGFP+ LV (green) area compared to Dylight 594+ BV area (red) in serial immunofluorescence images of each tissue. LV morphological changes that occurred in the post‐TPS course were quantified and compared as the ratio of LV length to pulp length by measuring the total length of the pulp and LVs in the 3D reconstructed images. Image J (v1.53a) was used for both analyses.

### Statistics

Levene and Shapiro–Wilk tests verified variance equality and normality. Tukey–Kramer or Kruskal‐Wallis followed by Steel‐Dwass test were used for multiple comparisons. Statistical analysis was done using R 4.0.1 (R‐Project, Vienna, Austria). *p*‐values below .05 were significant.

Note that detailed information on animals, randomization, sample size, experimental procedures, antibody suitability, primers used, and partial raw data can be found in Data S1 and Data S2.

## RESULTS

### Development of Prox1‐eGFP+ LVs in dental pulp

**FIGURE 2:**
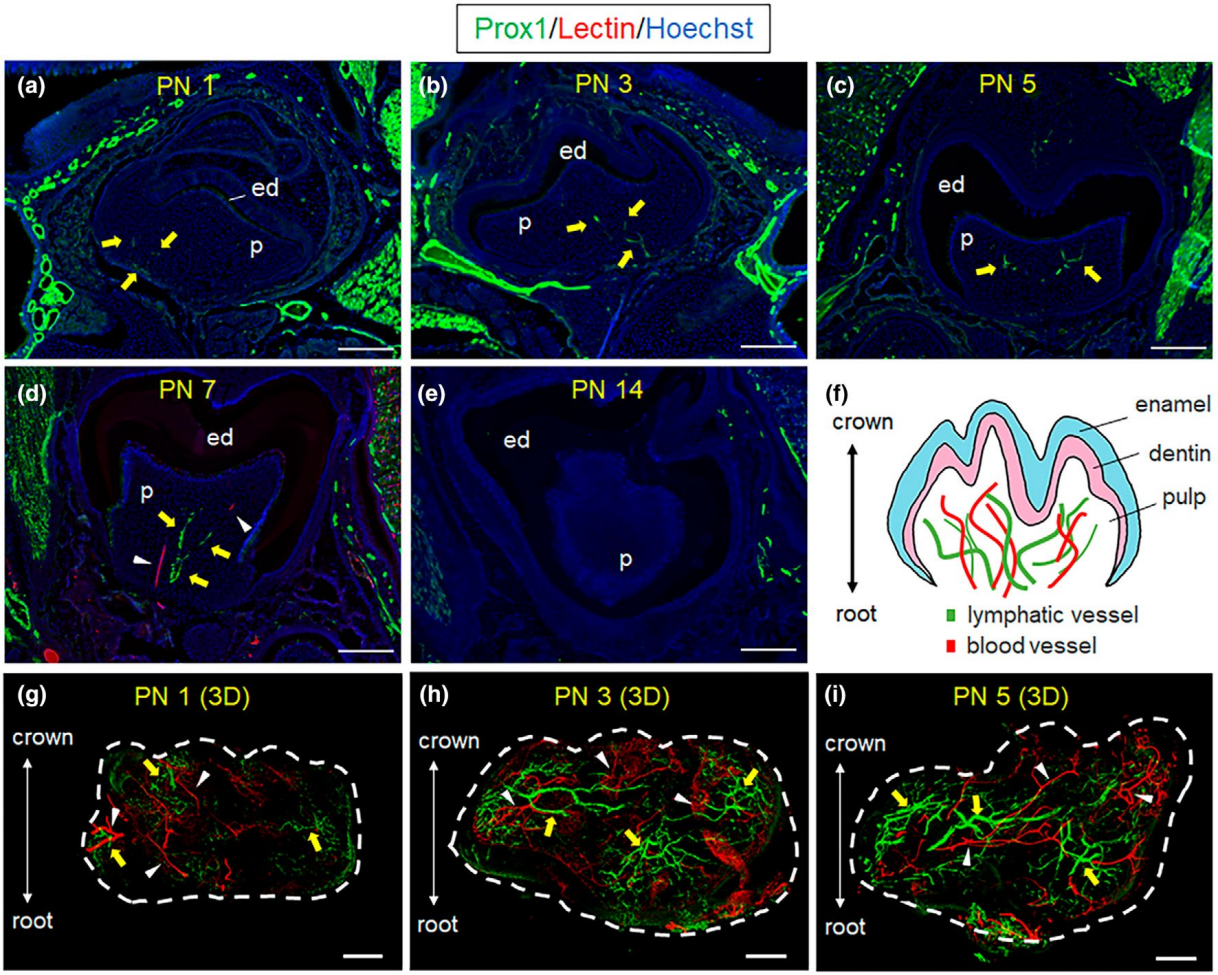
Abundant Prox1‐eGFP+ LVs independent of BVs are present in developing teeth prior to root formation. The distribution of Prox1‐eGFP+ LVs in developing tooth was examined by conventional immunofluorescence staining in frozen sections (a–e, *n* = 3 each) and 3D‐whole‐mount staining (g–i, *n* = 3 each). The schema shows the structures in tooth germ (f). (a, g) On postnatal day (PN) 1, a few Prox1‐eGFP+ structures (yellow arrows) were observed in the pulp, whilst the tooth germ was surrounded by many Prox1‐eGFP+ LVs. (b, h) The Prox1‐dependent eGFP signal increased at PN 3, indicating active development of the pulp lymphatics. LVs appeared to sprout into the pulp at PN3 (yellow arrows). (c, d, i) At PN 5, the development of a lymphatic network (green) independent of the vascular network (red) in the pulp is evident. At PN 7, developed LVs (yellow arrows) and BVs (white arrowheads) could be observed separately, even in a sliced specimen. LVs surrounding the tooth germ are less prominent at this stage compared to PN1. (e) However, on PN 14, in the early stage of root formation, Prox1‐eGFP+ LVs are absent from the pulp. Yellow arrows: Prox1‐eGFP+ LVs in dental pulp; White arrowheads: Lectin‐labelled BVs; ed., Enamel and dentin; p, Dental pulp; Bar: 200 μm.

First, we evaluated the development of Prox1‐eGFP+ vessels in mouse dental pulp during tooth development (Figure 2a–i). On postnatal day (PN) 1, we observed a few vessel structures with a weak Prox1‐eGFP signal in the dental pulp (Figure 2a,g). The number of Prox1‐eGFP+ vessels and the intensity of their eGFP signal increased with tooth development from PN 3 to PN 7 (Figure 2h,i). We observed all BVs by intracardially injecting Dylight 594‐conjugated tomato lectin, revealing that Prox1‐eGFP+ vessels did not overlap with lectin‐labelled BVs (Figure 2d,g–i). However, on PN 14, Prox1‐eGFP+ vessels were no longer observed in the dental pulp (Figure 2e). Interestingly, 3D whole‐mount imaging allowed us to observe a well‐developed Prox1‐eGFP+ vessel network on day 5, which is entirely independent of lectin‐labelled BVs (Figure 2i; Video S1).

### Inconsistency of pulpal Prox1‐eGFP+ LVs in mature teeth

**FIGURE 3:**
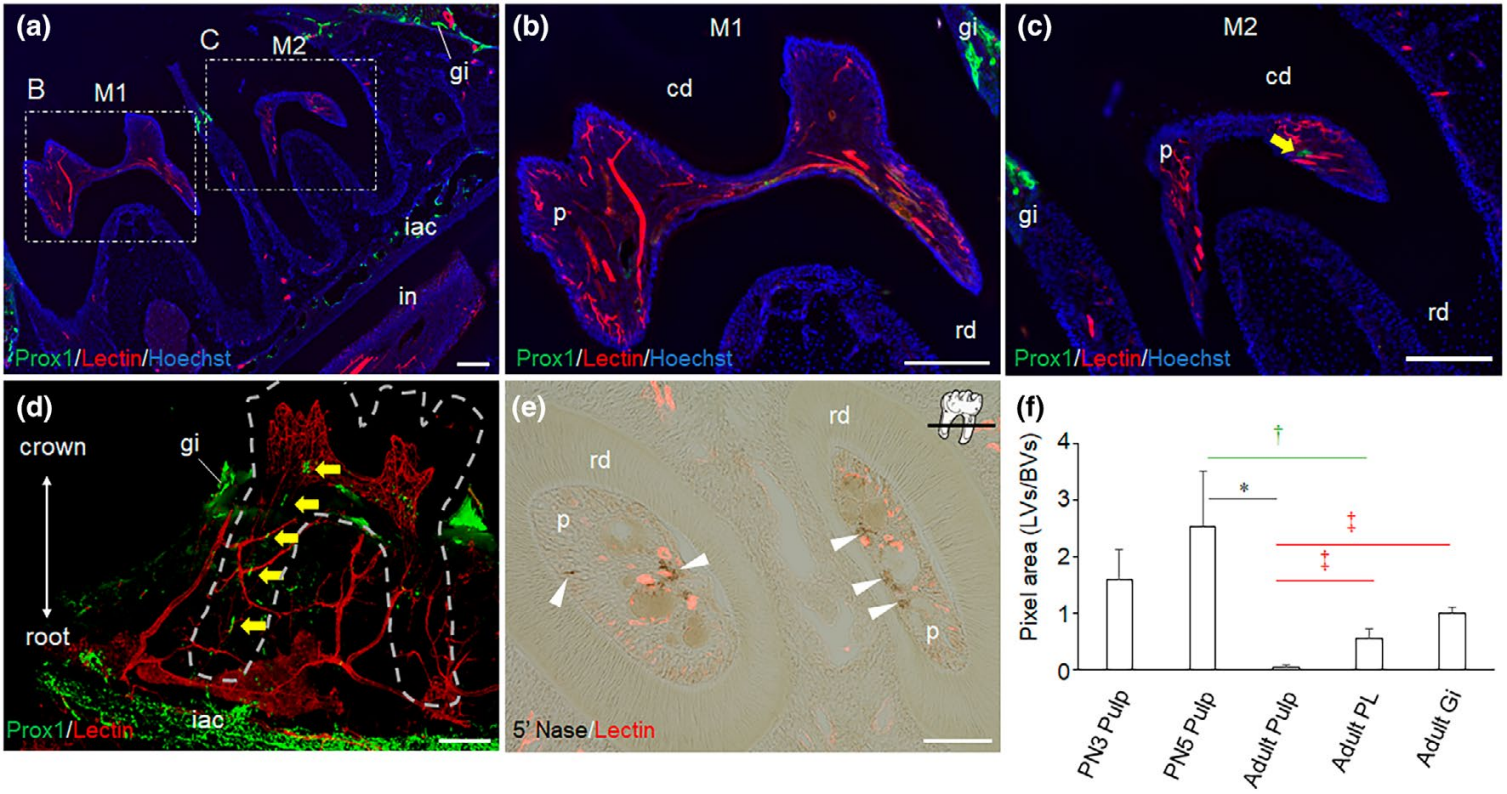
Pulpal LVs are few and difficult to detect in mature teeth. The distribution of pulpal LVs in adult mice mandible was examined by conventional immunofluorescence staining (a–c, *n* = 3), 3D whole‐mount staining (d, *n* = 3), or enzyme histochemistry (e, *n* = 3). (a–c) Well‐developed Prox1‐eGFP+ LVs are present in gingiva (gi) and inferior alveolar canal (iac), whereas the presence of LVs in mature dental pulp (p) is inconsistent. The boxed regions are magnified in (b) or (c). No Prox1‐eGFP+ LV is observed in the first molar (M1), whilst a small Prox1‐eGFP+ structure independent of BVs is observed in the second molar (M2). (d) 3D‐whole‐mount imaging reveals the distribution of Prox1+ LVs and vascular network in the mandible of an adult mouse. In the mature dental pulp, only a few scattered Prox1+ LVs with collapsed lumens are observed, whilst outside the pulp, especially in the iac, numerous Prox1+ LVs with well‐developed lumens and large diameters are observed. The white dotted line indicates the crown‐root outline. (e) 5′‐nucleotidase (5′‐Nase), considered a specific LV marker, was used for enzyme histochemistry. Shown is the presence of 5′ Nase+ structures (brown) distinct from lectin‐labelled BVs (red) in the horizontal mouse tooth section. The section position is indicated by a bar at the top right. 5′ Nase+ structures are mainly distributed around lectin‐labelled BVs. (f) LV density in dental pulp and periodontal tissues. The LV density in mature dental pulp was separately compared with that in different tooth development stages (postnatal days 3 and 5) and also with mature periodontal tissues. Well‐developed LV were present throughout tooth growth; however, in mature dental pulp, LV regression occurred and the density of LV markedly decreased (*p* < .05 vs. PN 5). The density of LV on mature pulp was significantly lower than periodontal soft tissue, including gingiva (*p* < .0005) and periodontal ligament (PL, *p* < .05). Incidentally, the LV density in the tooth germ at PN 5 was significantly higher than that in the PL (*p* < .05). * and ‡: *p* < 0.05 by Tukey–Kramer test, †: *p* < 0.05 by Student's *t*‐test; Error bar: Standard deviation. *n* = 3 each. Yellow arrow: Prox1‐eGFP+ LVs in dental pulp; White arrowhead: 5′ Nase+ structures; cd, Crown dentin; in, Incisor. Bar: 200 μm (a–c), 300 μm (D), 100 μm (e).

In adult mice, we observed numerous Prox1+ vessels with well‐developed lumens and large diameters in the gingiva and inferior alveolar canal (Figure 3a). However, the presence of Prox1‐eGFP+ vessels in the pulp of adult mice was inconsistent, even amongst mice born to the same parents (data not shown). Interestingly, within the same individual, Prox1‐eGFP+ vessels were not observed in the first molars, but were sometimes detected in the second molars, albeit this was not consistent (Figure 3b,c). 3D‐whole‐mount imaging revealed only a few scattered Prox1‐eGFP+ vessels with collapsed lumens in the mature tooth (Figure 3d; Video S2). On the contrary, enzyme‐histochemical staining for 5′‐Nase (a specific LV marker) (Kato et al., 2006) revealed the presence of 5′‐Nase+ vessel‐like structures in dental pulp. These 5′‐Nase+ structures appeared to be independent of Dylight 594‐lectin (Figure 3e). In contrast, other lymphatic markers, such as Vegfr3 and Lyve‐1, did not provide evidence for LVs in adult murine dental pulp (data not shown). In adult mice, the density of pulpal LVs normalized to BVs was significantly lower than that of the gingiva and periodontal ligament (PL). In contrast, the density of pulpal LVs in the tooth germ was higher than in mature pulp, especially at PN 5 with a significant difference (*p* < .05) (Figure 3f; Data S2), suggesting the low LV density is due to lymphatic regression that occurs during tooth development.

### Transient reappearance of Prox1‐eGFP+ LVs in stimulated pulp

**FIGURE 4:**
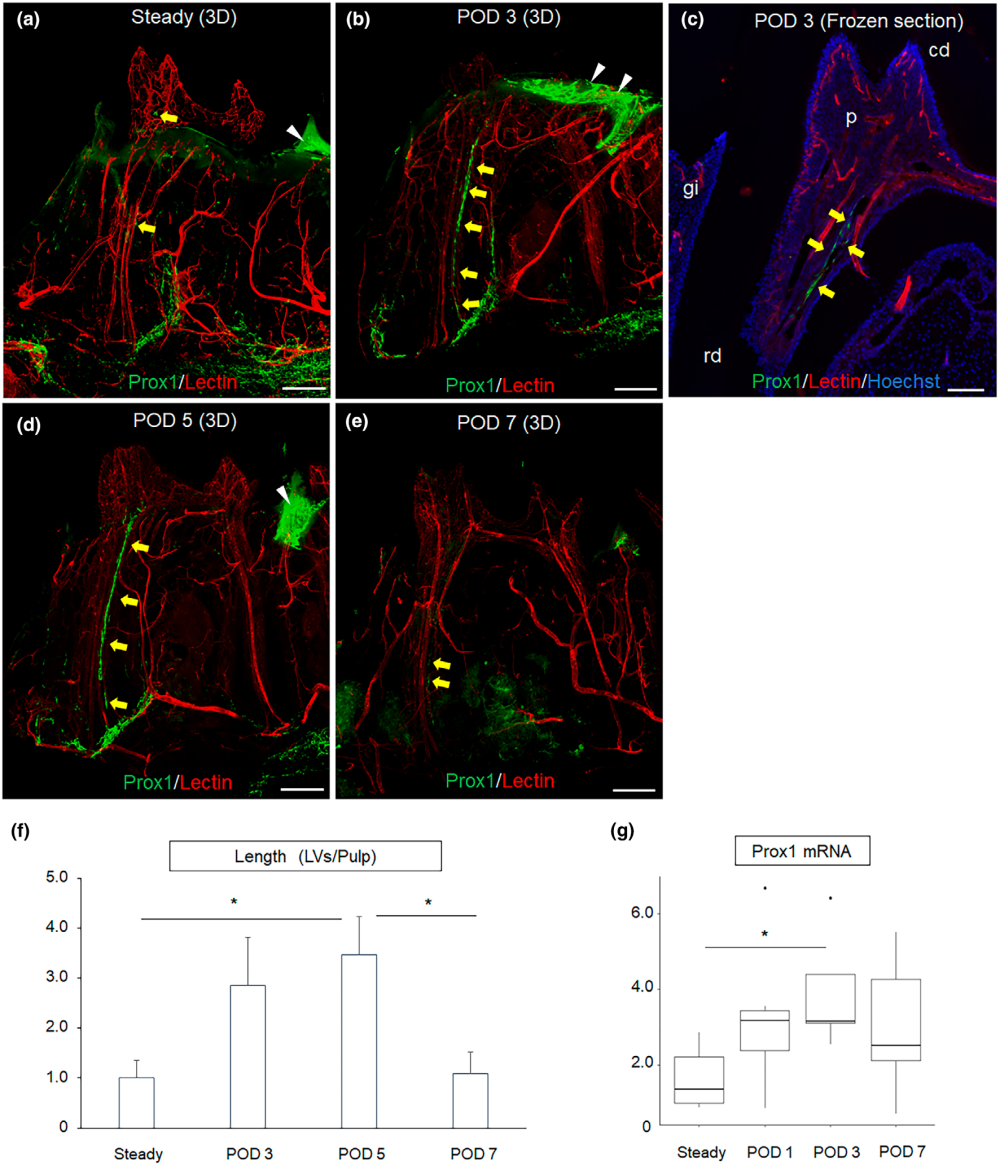
Transient induction of Prox1‐eGFP expression is observed in stimulated dental pulp. The dynamic change of pulpal Prox1‐eGFP+ LVs (green) after shallow cavity preparation was examined by 3D whole‐mount staining (a, b, d, and e) and conventional histology (c). (a) In the steady state, only a few discontinuous Prox1‐eGFP+ LVs (yellow arrows) are identified, scattered in the dental pulp (*n* = 3) (b) On postoperative day (POD) 3, Prox1‐eGFP+ LVs (yellow arrows) independent of lectin‐labelled BVs were observed with continuous lumens reaching the root apex, communicating with LVs in the periodontal ligament (*n* = 3). (c) In conventional immunofluorescence sections, Prox1‐eGFP expression is clearly observed in lymphatic endothelial cells (LECs) in enlarged LVs at POD3 (yellow arrows) (*n* = 3) (d) The status of LVs was similar on POD 5, showing continuity reaching the root apex (*n* = 3) (e) On POD 7, when the effect of pulp stimulation is thought to have subsided (Kieu et al., 2022), the distribution pattern, number and lumen diameter of Prox1‐eGFP+ LVs (yellow arrows) returned to that of the steady state (*n* = 3). (f) Morphological changes of LVs were compared by the ratio of LV length to pulp length in stimulated pulp; TPS increased the LV length ratio, reaching a peak at POD 5 (*p* < .05 vs. steady state, Tukey–Kramer test). Subsequently, the ratio decreased significantly and was comparable to the steady state on POD 7. Error bars: Standard deviation. (*n* = 3 each). (g) Kinetics of *Prox1* mRNA expression in the stimulated rat dental pulp. *Prox1* mRNA expression transiently increased, peaked on POD 3, then decreased on POD 7. The expression of *Prox1* mRNA normalized by *Actb* (beta‐Actin) is presented with a box plot. The horizontal bar inside the box indicates the median value. Statistical difference was tested by Kruskal‐Wallis followed by the Steel Dwass test (control: *N* = 9, POD 1: *N* = 8, POD 3: *N* = 5, POD 7: *N* = 7). Yellow arrow: Prox1‐Egfp+ LVs in dental pulp; White arrowhead: Remaining gingiva with Prox1‐eGFP signals; Bar: 200 μm (a, b, d, e); 100 μm (c); *p < 0.05.

In adult mice, it is known that inflammation can induce lymphangiogenesis (Liao & von der Weid, 2014). In mature dental pulp, Prox1‐eGFP+ LVs are small in diameter, discontinuous, and few in number. We hypothesized these LVs may increase in number and diameter during inflammation, making them easier to detect. To test this, we induced TPS by making a shallow cavity in the mandibular first molars. As before, in the absence of TPS, only a few scattered Prox1‐eGFP+ vessels were identified in dental pulp (Figure 4a). However, on postoperative day (POD) 3, we observed Prox1‐eGFP+ vessels independent of BVs with continuous lumens reaching the root apex (Figure 4b). The conventional sectional immunofluorescence staining at POD 3 also showed the presence of Prox1‐eGFP+ vessels with widely enlarged lumens and thin walls in the pulp (Figure 4c). A distinct large LV in the dental pulp was found to communicate with that in the PL (Figure 4b; Video S3). The status of LVs was similar on POD 5 (Figure 4d; Video S4). There were no signs of lymphangiogenesis. However, on POD 7, the LV distribution pattern and lumen diameter returned to that of the steady state, and the LVs were less readily identified as the inflammation resolved (Figure 4e; Video S5). The continuous length of pulpal LVs was transiently increased after cavity preparation, reaching a peak at POD 5. Its level was significantly higher than the steady state (Figure 4f), indicating that TPS restored lymphatic structures. Separately, *Prox1* mRNA expression was examined in rat dental pulp. Similar to eGFP expression in mouse teeth, it transiently increased after cavity preparation, peaked at POD 3, and decreased at POD 7 (Figure 4g).

### Evidence for drainage of interstitial fluid through the cell‐free zone and LV

**FIGURE 5:**
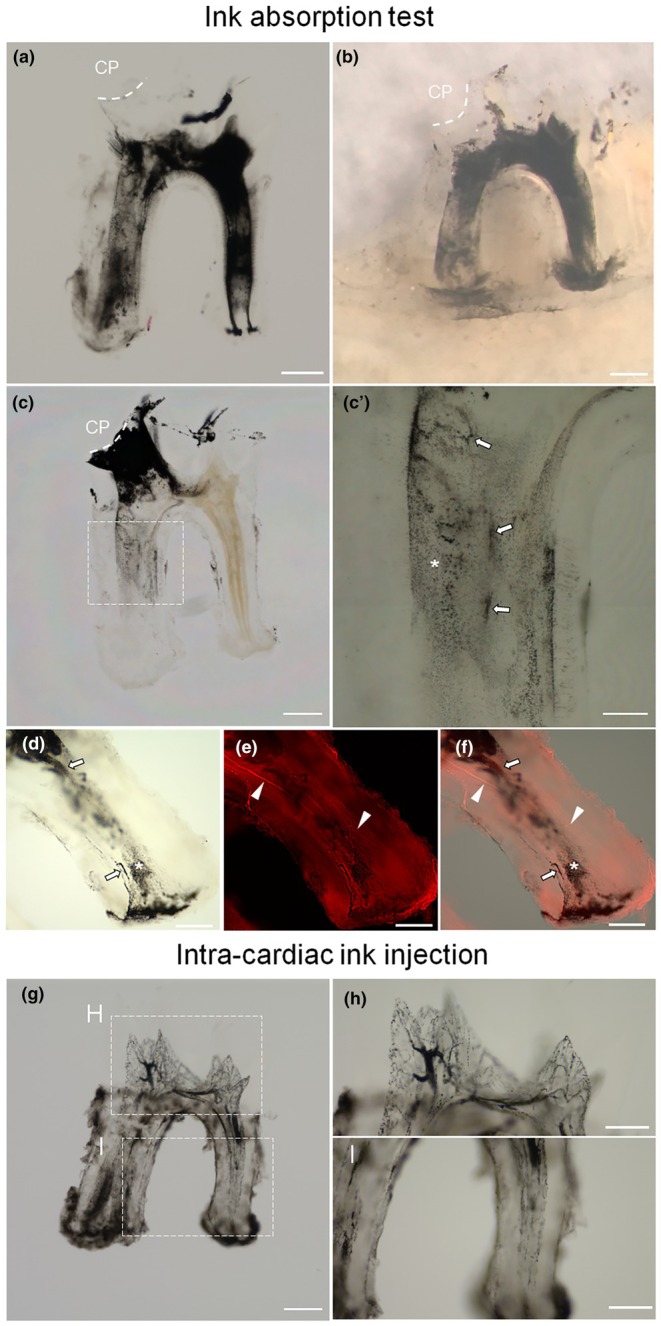
India ink applied to exposed pulp was distributed through dental pulp in the interstitial space and vessel‐like structures independent of BVs. Microscopic images of cleared mouse first molar/mandible subjected to ink absorption test are shown (a–c). 3D imaging of vascular network and ink particle distribution in the root of the first molar is presented (d–f). The 3D vascular network shown by intracardial ink injection is depicted (g–i). (a) India ink (1 μL) was applied on the exposed pulp of the first molar, causing the absorbed ink to diffuse throughout the pulp. (*n* = 3). (b) Ink particles reached the root apex and exuded from the apical foramen, with some being incorporated into the inferior alveolar canal (*n* = 3). (c) Diluted ink (10‐fold dilution, 1 μL) applied to the exposed pulp of the mandibular first molar. The location of ink particles indicates the interstitial drainage pathway in the dental pulp. The boxed region is magnified in c′. (*n* = 3) (c') Some of the ink particles were incorporated into vessel‐like structures (arrows), whilst most others were distributed in interstitial spaces (asterisk). (d–f) Ink particles are observed in interstitial space (asterisk) and vessel‐like structures (arrows). However, no ink particle was observed in lectin‐labelled BVs (red, arrowheads). Figure (d) and (e) were merged in Figure (f). (*n* = 3) (g) Ink particles were observed only in the vascular vessels, and there was no leakage from the vascular lumen. The boxed regions are magnified in H and I, showing the crown and root portions, respectively (*n* = 3). CP, Cavity preparation; Bar: 300 μm (a–c, g); 200 μm (d–f); 100 μm (h, i).

Although we identified LV in mouse dental pulp, it remained unclear if these vessels function as a drainage pathway for pulpal interstitial fluid. To investigate this, we tested the lymphatic uptake of Indian ink using a tissue‐clearing technique. When applied to exposed pulp, the ink diffused throughout the dental pulp (Figure 5a,b), and in some of the samples, it reached the inferior alveolar canal through the apical foramen (Figure 5b). We then applied a 10‐fold diluted ink to the exposed pulp to reveal detailed drainage pathways. Notably, the ink particles were widely distributed in interstitial spaces but were sometimes identified in vessel‐like structures as clusters (Figure 5c,c′). No ink particle was absorbed by Dylight 594‐lectin‐labelled BVs (Figure 5d–f). We observed no extravascular leakage of transcardially injected ink, negating the vascular permeability of the ink particles. Furthermore, the aspect of the vascular network (Figure 5g–i) was clearly different from that of the distribution of ink particles shown in Figure 5c, suggesting the presence of drainage pathways other than BVs in the mouse dental pulp.

**FIGURE 6:**
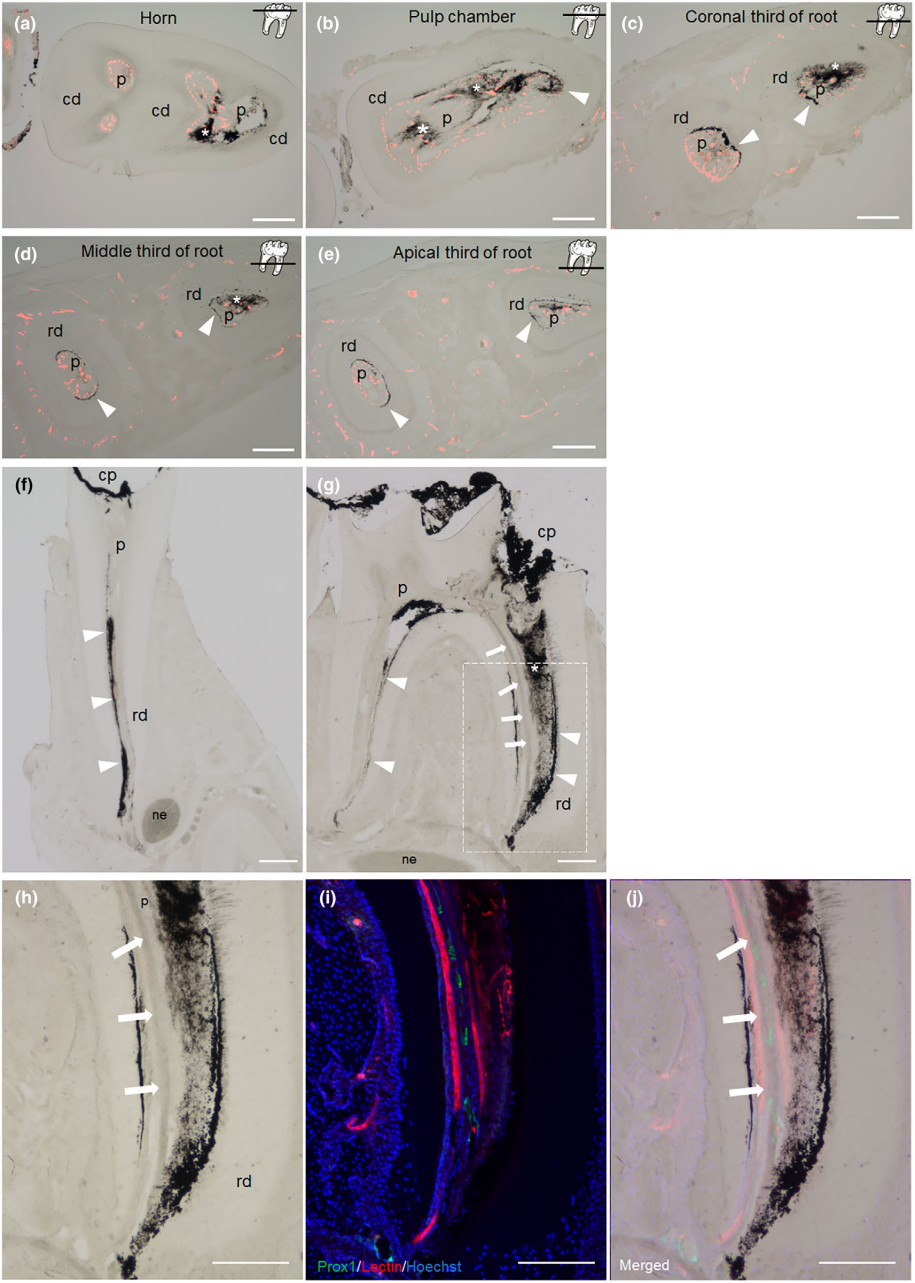
Mouse dental pulp probably utilizes two interstitial fluid drainage pathways: The extravascular pathway and LV. The mandibular first molar in steady state (a–e) and in TPS at POD 3 (f–j) were subjected to the ink absorption test. (a–e) A serial horizontal cross‐section of mouse mandible subjected to ink absorption test and intra‐cardiac injection of DyLight 594‐lectin. Ink particles were irregularly observed in interstitial spaces (asterisks) at the horn (a) and pulp chamber level (b). In contrast, at the root level, the localization of ink particles was mainly in cell‐free zone (arrowheads) (c–e). No ink particle was observed in lectin‐labelled BVs (red) in any section (*n* = 3). Raw images before merging are shown in Data S1. (f) The frontal section of the mandible subjected to ink absorption test. The absorbed ink was distributed through the cell‐free zone and reached the root apex (*n* = 3). (g) Sagittal section of an ink absorption test in the TPS study on day 3 post cavity preparation. Prox1‐eGFP mice were subjected to TPS by trimming the mesial surface. Three days after the operation, cavity preparation was made on the first molar again to expose dental pulp and India ink applied to exposed the dental pulp. Ink particles were distributed in vessel structures (arrows), interstitial spaces (asterisk) and cell‐free zone (arrowheads) (*n* = 3). (h–j) High magnification of the area framed in Figure g (h). The immunofluorescence image of the same area as in Figure h (i). The composite image with brightfield and immunofluorescence image (j). Ink particles (black) were present in the vessel structure that express Prox1‐eGFP (green), and reach the root apex, suggesting that the pulpal LVs distinct from BVs (red), uptake ink particles to eject them out of the tooth. Cp, Cavity preparation; p, Pulp; cd, Crown dentin; rd, Radicular dentin; Bar: 200 μm.

To further clarify the detailed drainage pathway, we histologically examined the mandibular samples subjected to the ink absorption test. Horizontal sections revealed that the distribution pattern of absorbed ink was different depending on the pulp location. At the horn and pulp chamber level, ink particles were irregularly distributed in the interstitial space (Figure 6a,b). On the contrary, ink particles were localized mainly in the cell‐free zone at the level of the coronal third to apical third of the root (Figure 6c–e). No accumulation of ink particle is observed in lectin‐labelled vessels in any section. The frontal plane section confirmed that absorbed ink was distributed in the interstitial space, mainly in cell‐free zones (Figure 6f). We also examined the absorption of the ink in stimulated pulp on day 3 post cavity preparation; ink uptake into Prox1‐eGFP+ vessel was readily observed in addition to the interstitial space (Figure 6g,h). The Prox1‐eGFP vessel was distinguished from lectin‐labelled vessels (Figure 6i). Prox1‐eGFP signals were observed in a vessel structure filled with ink particle, (Figure 6j). These results suggest that pulp LV can act as a drainage pathway in the post‐TPS course.

## DISCUSSION

In this study, the dynamics of dental pulp LVs during tooth development and in transiently stimulated pulp were studied in detail using a combination of multiple lymphatic markers, imaging techniques, and a function test; our study reveals intriguing remodelling dynamics in LVs during tooth development and TPS. Pulpal Prox1+ vessels, abundant in early tooth development, drastically regress between 7 and 14 days of age, becoming almost undetectable in mature teeth, although there are no significant changes in the BV network (Yoshida et al., 1988). However, fragmented Prox1+ LVs exhibited contiguous structures in response to TPS, connected to LVs in the PL, and are involved in interstitial fluid drainage. In a clinical context, the main cause of pulpitis is caries (infection‐induced pulpitis). In contrast, short‐term stimuli, such as invasive tooth preparation, even non‐infectious, induce a brief acute inflammation, which involves damage‐associated molecular patterns that are recognized by pattern recognition receptors, triggering events similar to those caused by pathogens and pathogen‐associated molecular patterns likely (Pohl et al., 2024). In both infectious and non‐infectious conditions, when the stimulus/source of stimulus is removed at the appropriate time, inflammation then resolves, followed by tissue regeneration or repair. Thus, we can assume that even in the case of infection‐induced reversible pulpitis, LV dynamics similar to those of non‐infectious TPS occur, with transient Prox‐1 expression at the onset, then disappearing during the resolution.

The presence and function of LVs in the dental pulp have been a topic of long‐term debate. Recent studies have provided conflicting evidence for the presence of LVs in dental pulp. Wiśniewska et al. (2021, 2022) suggest a correlation between pulpitis and the formation of new vessels, including LVs, but do not conclusively demonstrate their presence. In contrast, Lohrberg (Lohrberg & Wilting, 2016) and Berggreen (Berggreen et al., 2020) state that LV are absent from dental pulp in mice based on immunohistochemistry using lymphatic markers. Ultrastructural observations by electron microscopy have shown the presence of lymphatic vessel‐like structures in the dental pulp (Marchetti & Poggi, 2002). However, given the previous studies elucidating the expression of various lymphatic markers (Berggreen et al., 2020; Lohrberg & Wilting, 2016), it is inconclusive that functional pulp lymphatic vessels exist.

Definitive identification and visualization of LVs in the pulp have been challenging and confounded by the choice of lymphatic markers used in studies, and until recently, the lack of availability of optimized visualization methods for this tissue. Lymphatic markers such as Lyve‐1, Prox1, and Vegfr3 are frequently used to identify LVs (Baluk & McDonald, 2008; Kong et al., 2017), but there are many points to note, such as the expression of Lyve‐1 on pulp resident macrophages (Berggreen et al., 2009; Kieu et al., 2022) and the discrepancies in reports of the presence (Berggreen et al., 2009; Takahashi et al., 2012) or absence (Gerli et al., 2010) of pulp Vegfr3+ vessels. In addition, LVs do not always express all of these markers, and the expression status of markers varies from tissue to tissue (Fujimoto et al., 2020; Takeda et al., 2019).

Prox1‐dependent eGFP was used as the primary lymphatic marker in this study and separated from DyLight 594‐lectin‐labelled blood vessels. As the eGFP signal declined with tooth development, enzymatic histochemical staining for 5′‐Nase, a lymphatic marker (Kato et al., 2006), was used in conjunction with gene expression analysis. Visualization was performed with 3D imaging using the PEGASOS tissue‐clearing method. In addition, we used regular histochemistry and immunofluorescence staining of tissue sections. As a result, LVs in the pulp were detected more clearly in samples with 3D whole‐mount staining. This is probably because the LVs to be detected fragmented in thin sections, whereas the structure of the LVs was maintained in the whole‐mount sample, improving signal detection.

In time‐passed cadavers and resected specimens, absorption of India ink does not occur due to functional disruption of LVs (Sato et al., 2021). In contrast, under deep anaesthesia, ink particles could be absorbed into LVs. Also, based on the property that particles larger than 10 nm in diameter are preferentially taken up via the LV, whilst particles smaller than 10 nm in diameter are absorbed via the BV (Ali Khan et al., 2013), the draining function of LVs was examined using India ink. This classical method (Clark et al., 2010) revealed that the drainage pathways of interstitial fluid in the dental pulp include lymphatic and extravascular pathways. Despite the limitations of technical difficulty, the limited amount of ink that can be applied to the pulp, and the inability to completely exclude the effects of micro wounds during pulp exposure, this approach could be applicable to trace the functional drainage pathway of interstitial fluid from the pulp.

Our finding, regression of pulp LV, needs to be carefully considered whether this is similar to LV regression related to organ function, such as in the cornea (Zhong et al., 2017). Interestingly, the timing of LV disappearance in the pulp coincides with key developmental milestones: initiation of root formation and tooth eruption. The drainage of pulpal interstitial fluid must be governed by a tissue pressure gradient (driving pressure) (Berggreen et al., 2020). In other words, interstitial fluid flows passively and is drawn into LVs by external forces, such as pulsatile artery pressure and vibration. Foreign substances also enter LVs this way. External forces to the LVs also drive peripheral lymph transport. Indeed, in the ink absorption test, when the suction tip was placed on the second molar and negative pressure was applied, the ink on the exposed pulp was slowly absorbed into the pulp (data not shown). The passive process above, rather than natural diffusion, is thought to be the major mechanism by which India ink enters vital pulp and pulp LVs. The tooth germ is not influenced by external pressure as it is enclosed by bone; in this phase, the pulpal interstitial fluid drainage likely depends on LVs and arterial pulsation. As the tooth grows, it migrates from the interosseous to the submucosa and encounters new functional pressures (primarily occlusal forces) from the oral cavity during the eruption. Experimental forces equivalent to physiological occlusal forces have been found sufficient to squeeze fluid out from the pulp to the outside of the tooth (Paphangkorakit & Osborn, 2000). Also, our India ink experiments strongly suggest the cell‐free zone as an alternative extravascular route for interstitial fluid drainage, backed by the concept of fluid flow through loose interstitium (Berggreen et al., 2020; Heyeraas, 1989; Oehmke et al., 2003). Taken together, we speculate that the regression of LVs in the dental pulp is related to a decrease in the functional demands of the LVs due to changes in the primary driving pressure during the tooth development process.

When the host, including dental pulp, is exposed to harmful stimuli, the innate immune response is initially triggered. The nature of the stimulus (infectious or non‐infectious) and its persistence are then assessed, leading to the induction of a subsequent inflammatory response. Beyond fluid drainage, LVs serve as vital pathways for immune cells in this process, especially antigen‐presenting cells in the early stages of the immune and inflammatory response. The absence of lymphatic vessels may lead to inefficient antigen‐presenting cell migration, even with the function of the extravascular pathway. However, considering the rarity of pulpitis due to protection by sound tooth structure in the physiological state, these vessels may enter a stage of functional quiescence characterized by low activity and non‐proliferating endothelial cells. Integration of transduction signals and transcriptional regulation is required to restore the quiescent LECs (Tabrizi et al., 2021). Prox1 has been suggested as a multifaceted cell cycle regulator in various cell types (Baxter et al., 2011) besides being the prominent marker of LECs; the presence or absence of Prox1 expression in the G0/G1 phase seems to determine the quiescent state (Dyer et al., 2003) or transition to the S phase (Baxter et al., 2011). Therefore, we propose that pulpal LVs apparently regress due to arrested Prox1 expression and are reactivated and re‐functional upon re‐expression of Prox1 in response to inflammatory stimuli. It is reasonable to consider TPS as a form of sterile inflammation induced by various stresses associated with cavity preparation, but not infection. Given the critical roles of the damage‐associated molecular pattern–pattern recognition receptor axis and downstream inflammatory mediators in the sterile inflammation (Gong et al., 2020), we expect that these factors are expected to be involved in lymphatic reactivation. However, this study did not elucidate the details underlying the functional quiescence of LVs and the reactivation mechanism of pulpal LVs. Future integrated multidisciplinary studies that address the above issues are needed to understand the comprehensive characteristics of pulpal LV and to lead to improved treatments for pulpitis.

## CONCLUSION

Lymphatic vessels are present in the dental pulp of mice. Remodelling of LVs progresses with tooth development. In mature dental pulp, LVs are difficult to detect as they are likely in a quiescent state, including a marked decrease in Prox1 expression. However, the quiescent LVs can be reactivated by inflammatory stimulation. India ink‐based imaging revealed that there are two functional interstitial draining routes in the pulp: the LVs and the extravascular interstitial draining pathway.

## AUTHOR CONTRIBUTIONS

KT and HS: Contributed to conception, design, data acquisition and interpretation, performed all statistical analyses, drafted and critically revised the manuscript. DC, AF, PK: Contributed to conception, design, data acquisition and interpretation, drafted, and critically revised the manuscript. All authors gave final approval and agreed to be accountable for all aspects of the work.

## FUNDING INFORMATION

The authors acknowledge the following financial support for the present study: grants from NIH/NIDCR (R21DE030213 to HS and PK, R01DE024796 and R56DE024796 to HS), NIH/NHLBI (R01HL120888 to PDK), and a TMDU priority research area grant (3301019207 to KT).

## CONFLICT OF INTEREST STATEMENT

The authors have no conflicts of interest to declare.

## ETHICS STATEMENT

All animal studies were approved by the Animal Care and Use Committee at the Tokyo Medical and Dental University (TMDU, A2023‐196C3) and the University of Michigan (UM, #PRO00009610). Note that the TMDU has been recently reorganized as Science Tokyo. All animal experiments were conducted in compliance with the Preferred Reporting Items for Animal Studies in Endodontology (PRIASE) 2021 guidelines.

## Supporting information


Data S1.

Data S2.

Video S1.

Video S2.

Video S3.

Video S4.

Video S5.

## Data Availability

The datasets that support the findings of the present study are available from the corresponding author upon reasonable request.
