# Supplementary material for: Dental pulp lymphatic vessel dynamics during tooth development and pulp stimulation in rodents
Source: Int Endod J. 2025 Apr 25;58(8):1197–210. doi: 10.1111/iej.14244 (PMC12254533; doi:10.1111/iej.14244)
Supplement: Supplementary file 1 — Data S1. [file IEJ-58-1197-s006.docx]

**Supplemental Information**

Title: Dental pulp lymphatic vessel dynamics during tooth development and pulp stimulation in rodents

**Details of Materials and Methods**

**Use of animals**

**Appendix Table 1.** **Detailed information of animals used in this study.**

| **Species/strains** | **Age** | **Purpose** | **Groups** | **N=** |
| --- | --- | --- | --- | --- |
| Prox1-eGFP mice | 6-8 weeks | 2D IHC | None | 3 |
|  | PN 1* | 2D IHC | None | 3 |
|  |  | 3D whole mount | None | 3 |
|  | PN 3 | 2D IHC | None | 3 |
|  |  | 3D whole mount | None | 3 |
|  | PN 5 | 2D IHC | None | 3 |
|  |  | 3D whole mount | None | 3 |
|  | PN 7 | 2D IHC | None | 3 |
|  | PN 14 | 2D IHC | None | 3 |
|  | 5 weeks | Ink absorption 2D | None | 9 |
|  | 5 weeks | Ink absorption 3D | None | 12 |
|  | 5 weeks | Ink intra-cardiac injection 3D | None | 3 |
|  | 5 weeks | Pulp stimulation | control | 3 |
|  |  |  | POD 3⁑ | 6 |
|  |  |  | POD 5 | 3 |
|  |  |  | POD 7 | 3 |
| SD rats | 8 weeks | Gene expression analysis | control | 9 |
|  |  |  | POD 1 | 8 |
|  |  |  | POD 3 | 5 |
|  |  |  | POD 7 | 7 |

*PN: Postnatal day

⁑POD: Postoperative day

Note that animals subjected to pulp stimulation experiments were randomly assigned to each group. The evaluation of LV during aseptic reversible pulpitis was carried out in a single blinded manner so that an examiner was not informed about group settings. The sample number of gene expression analysis was based on power analysis and our preliminary studies (data not shown).

**2D-Immunohistochemistry**

Mandibles of Prox1-eGFP mice were isolated, decalcified in 15% ethylenediaminetetraacetic acid, and subjected to immunohistochemistry (14 μm cryosections) as described (Tazawa et al. 2020). The eGFP signal was enhanced using a rabbit polyclonal anti-GFP antibody (1:500; NB600-308; Novus Biologicals, Centennial, CO; at 4°C for overnight) and Alexa Fluor 488- or 594-conjugated goat anti-rabbit secondary antibody (1:500; ab150077 or ab150080; Abcam, Cambridge, MA; at room temperature for 60 minutes). The 4',6-diamidino-2-phenylindole, dihydrochloride was used for counterstaining. Images were captured with a BZ-X700 microscope (Keyence, Osaka, Japan).

**5’-Nase reaction solution for Enzyme-histochemical Staining**

The 5'-Nase reaction solution was obtained by mixing the following regents and solutions, then adding deionized water up to total 10 ml.

- 0.1 M Tris-maleate buffer (PH7.2, #T3128, Sigma-Aldrich, Burlington, MA): 4.0 ml
- Sucrose (#S0389, Sigma-Aldrich): 0.7 g
- 3.6 mM Magnesium sulfat (#AC413480050, Thermo Fisher Scientific, Waltham, MA): 0.4 ml
- 2.0 mM levamisole (#T121510G, TCI America, Portland, OR): 1.0 ml
- 3.6 mM Lead(II) nitrate (#AC315242500, Thermo Fisher Scientific): 0.6 ml
- 2.9 mM adenosine 5’monophosphate (# A2252, Sigma-Aldrich): 4.0 ml

**3D-Whole Mount Staining**

Mandibles and mandibular first molars isolated from Prox1-eGFP mice were subjected to 3D whole-mount observation. As the eGFP signal is diminished after clearing during the PEGASOS method (Jing et al. 2018), a signal recovery process using whole-mount staining with an anti-eGFP antibody and an immunofluorescent dye-conjugated secondary antibody was employed as previously described (Tazawa and Sasaki 2023). In brief, whole-mount immunolabeling was conducted between the decolorization and delipidation steps in the PEGASOS method. Samples were incubated with an anti-eGFP antibody (1:500) prepared in the staining buffer for 4 days at room temperature. After three washes in tris-buffered saline with Triton X-100, the samples were incubated with an Alexa Fluor dye-conjugated anti-rabbit antibody for 4 days at room temperature. Antibody information is as above. The Stellaris 5 confocal microscope (Leica Microsystems, Buffalo Grove, IL) was used for 3D imaging.

- Staining buffer: 10 mM HEPES (#2478776, Gibco, Carlsbad, CA), 10% (v/v) Triton X-100 (#T8787, Sigma-Aldrich), 200 mM sodium chloride (NaCl, #S9888, Sigma-Aldrich), 0.5% goat serum, and 0.05% sodium azide (#S2002, Sigma-Aldrich) in deionized distilled water (DDW).
- Blocking buffer: Staining buffer containing 10% goat serum.
- Tris-buffered saline with Triton X-100 (TBST) (pH 7.4): Dissolve 50 mM Tris hydrochloride (Trise–HCl, #15506017, Invitrogen, Waltham, MA), 150 mM NaCl (Sigma-Aldrich), and 0.1% (v/v) Triton X-100 (Sigma-Aldrich) in DDW.
- Decolorization solution: Dissolve 25% (v/v) Quadrol (#122262, Sigma-Aldrich) and 5% (v/v) ammonium solution (#105432, Sigma-Aldrich) in DDW.
- 30%/50%/70% tert-butanol (tB) gradients (pH > 9.5): Dissolve 30%/50%/70% (v/v) tB (#360538) and 3% (w/v) Quadol in DDW.
- tB-polyethylene glycol (PEG): Dissolve 70% (v/v) tB, 27% (v/v) poly(ethylene glycol) methyl ether methacrylate (PEGMMA)500 (#447943, Sigma-Aldrich), and 3% (w/v) Quadol in DDW.
- Benzyl benzoate (BB)-PEG: Dissolve 75% (v/v) BB (#B6630, Sigma-Aldrich), 25% (v/v) PEGMMA 500 (Sigma-Aldrich), and 3% (w/v) Quadol in DDW.

**Prox1 gene expression analysis**

Male Sprague Dawley rats (a total of 29 rats, CLEA Japan, Tokyo, Japan) were subjected to pulp stimulation and euthanized on days 0 (control), 1, 3, and 7. The first molars were isolated immediately and split at the cementoenamel junction. Coronal dental pulps were isolated and subjected to total RNA extraction (PicoPure™ RNA Isolation Kit, Applied Biosystems, Waltham, MA). The RNA samples (300 ng each) were subjected to cDNA synthesis (PrimeScript RT Master Mix, Takara, Shiga, Japan). The Prox1 mRNA expression level in the course of ARP was compared by quantitative RT-PCR using pre-designed primers for Prox1 and Actb, the TaqManTM Universal Master Mix II, with UNG (#4440042, Applied Biosystems), and the CFX96 cycler (Bio-Rad, Hercules, CA). Relative gene expression values were calculated as 2−ΔΔCT. The Smirnov-Grubbs test was used to detect outliers, and no outlier was detected.

**qRT-PCR – TaqMan Assay Primer information**

Pre-designed TaqMan Gene Expression Assays provided by Applied Biosystems (Waltham, MA) were used for gene expression analysis.

**Supplemental Table 2.** **Primers for rat genes used in this study.**

| **Genes** | **Assay ID** |
| --- | --- |
| *Actb* (b-actin) | Rn00667869_m1 |
| *Prox1* | Rn02103824_s1 |

**The quantitative analysis of tissue LV density**

For each serial immunofluorescence image, the outline of dental pulp was set in the Image J (v1.53a) and only the pulp area was extracted. The images were then divided into the three primary colours, and the red and green pixel areas within a threshold of 50-255 were measured. For each colour, the average pixel area in serial images was measured and the ratio of green to red was calculated to detect the LV density in the pulp. The raw data are found in Supplemental table 1.

**Supplemental Figure and Legend**


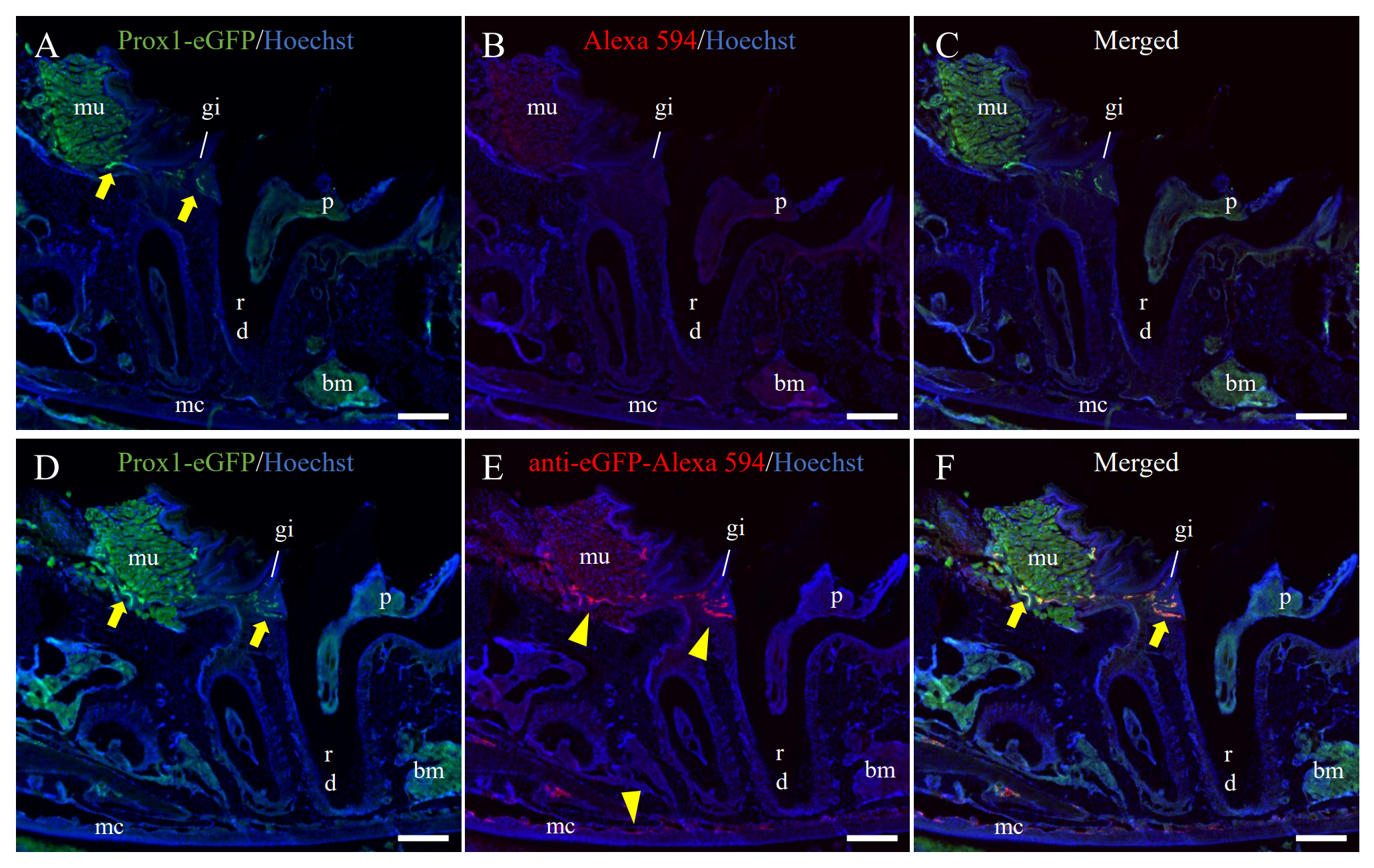


**Supplemental Figure 1.** **Appropriateness of Immunofluorescence Imaging.**

Indicated in (A-C) is minimal non-specific binding of an Alexa Fluor 594-conjugated goat anti-rabbit secondary antibody (Abcam, Cambridge, MA, ab150080). **(A)** The eGFP signal derived from Prox1-eGFP mice. **(B)** The represent signal produced by a secondary antibody (ab150080) in the absence of the primary antibody. **(C)** The image of the merged signals; no specific binding of the secondary antibody detected. Note that nuclei are labeled with DAPI (blue). Binding of primary antibody to mouse-derived eGFP and subsequent detection with secondary antibodies is shown in (D-F). **(D)** The eGFP signal produced by Prox1-eGFP rodents. **(E)** The signal represents evidence of secondary antibody binding to the primary antibody. **(F)** The merged image of these two signals; the secondary antibody signal (Alexa 594) co-localized virtually with the mouse-derived eGFP signal. Since the primary antibody used was a rabbit polyclonal antibody, a control antibody was not applicable. However, the images obtained show that the combination of antibodies used effectively detected and amplified the eGFP signal from mice.

gi: gingiva, rd: radicular dentin, p: pulp, mu: muscle, mc: mandibular canal, bm: bone marrow

Arrow: Prox1-eGFP+ vessel, Arrowhead: Alexa 594+ vessel Bar: 300 µm.


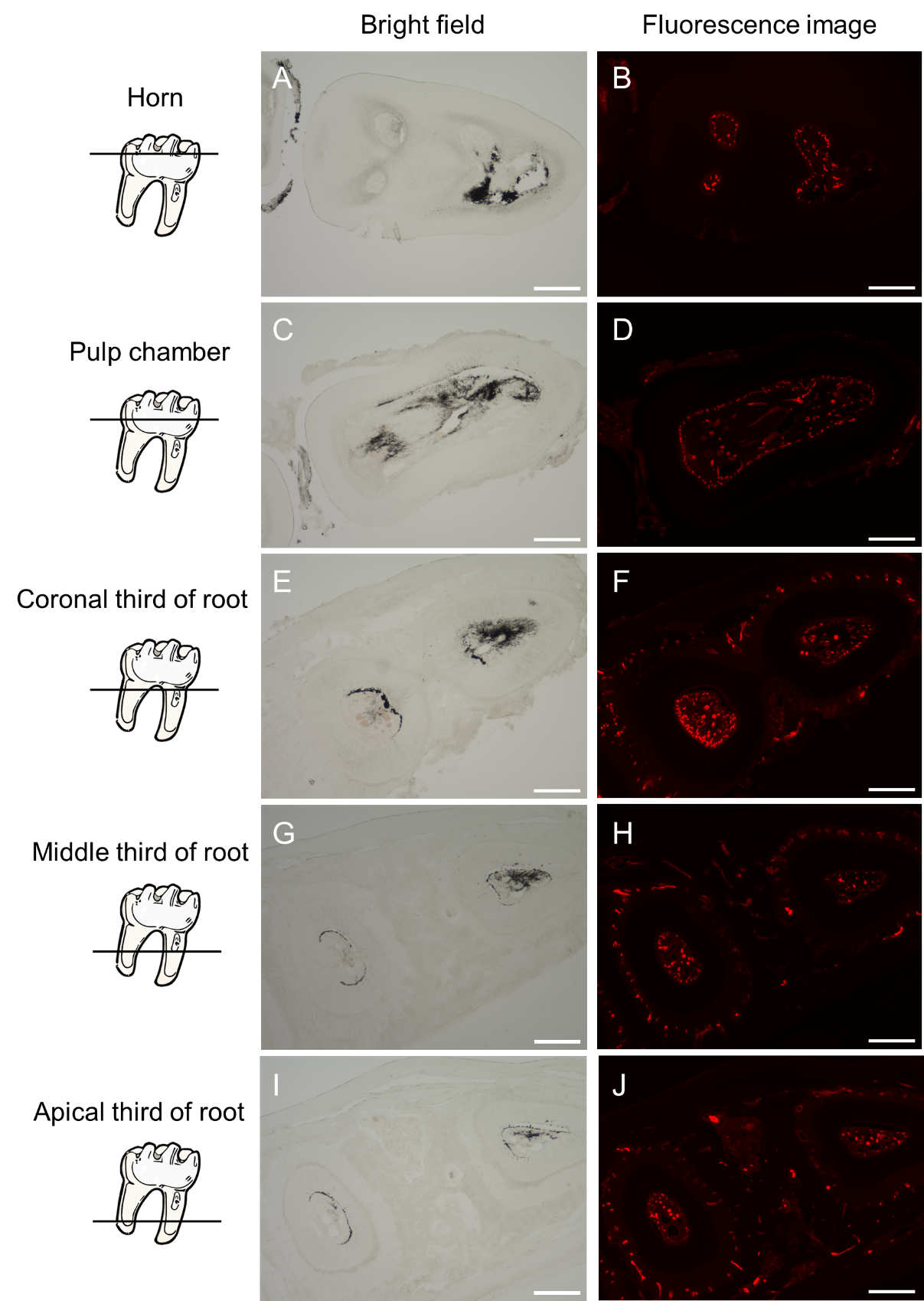


**Supplemental Figure 2. Bright field and fluorescence imaging in horizontal tooth sections subjected to ink absorption and blood vessel labeling.**

**(A, C, E, G, and I)** are bright field images showing the distribution of absorbed ink (black). **(B, D, F, H, and J)** are fluorescence images showing the location of blood vessels labelled with Alexa Fluor 594-conjugated lectin (red). **(A-J)** Horizontal cross sections at each of the positions shown in the schematic diagram on the left. Bar: 200 µm.

**Reference**

Tazawa K, Kawashima N, Kuramoto M, Noda S, Fujii M, Nara K, Hashimoto K, Okiji T. 2020. Transient receptor potential ankyrin 1 is up-regulated in response to lipopolysaccharide via p38/mitogen-activated protein kinase in dental pulp cells and promotes mineralization. Am J Pathol. 190(12):2417-2426.

Tazawa K, Sasaki H. 2023. Three-dimensional cellular visualization in mouse apical periodontitis using combined whole-mount staining and optical tissue clearing. J Oral Biosci. 65(1):132-135.

Jing D, Zhang S, Luo W, Gao X, Men Y, Ma C, Liu X, Yi Y, Bugde A, Zhou BO et al. 2018. Tissue clearing of both hard and soft tissue organs with the pegasos method. Cell Res. 28(8):803-818.
